# Dynamics of Aggregation in Systems of Self-Propelled Rods

**DOI:** 10.3390/e26110980

**Published:** 2024-11-15

**Authors:** Richard J. G. Löffler, Jerzy Gorecki

**Affiliations:** 1 Center for Star and Planet Formation, Globe Institute, University of Copenhagen, Øster Voldgade 5-7, 1350 Copenhagen, Denmark; richard.loffler@sund.ku.dk; 2 Institute of Physical Chemistry, Polish Academy of Sciences, Kasprzaka 44/52, 01-224 Warsaw, Poland

**Keywords:** matter aggregation, structure formation, cluster–cluster aggregation, camphene–camphor–polypropylene plastic, interfacial phenomena, surface tension, self-propelled motion, rods, weak vorticity analog

## Abstract

We highlight camphene–camphor–polypropylene plastic as a useful material for self-propelled objects that show aggregation while floating on a water surface. We consider self-propelled rods as an example of aggregation of objects characterized by non-trivial individual shapes with low-symmetry interactions between them. The motion of rods made of the camphene–camphor–polypropylene plastic is supported by dissipation of the surface-active molecules. The physical processes leading to aggregation and the mathematical model of the process are discussed. We analyze experimental data of aggregate formation dynamics and relate them to the system’s properties. We speculate that the aggregate structure can be represented as a string of symbols, which opens the potential applicability of the phenomenon for information processing if objects floating on a water surface are regarded as reservoir computers.

## 1. Introduction

**Figure 1:**
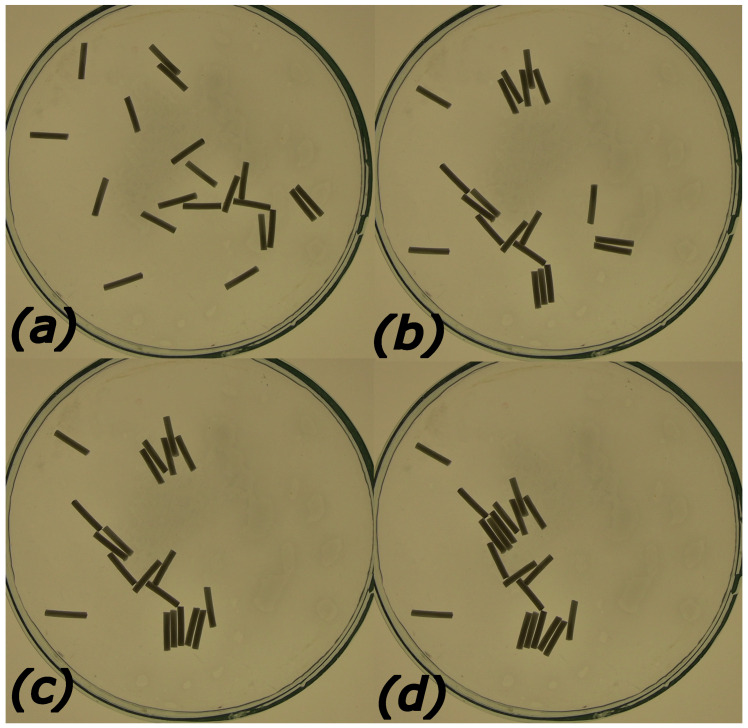
Aggregation in a system of polylactic acid rods driven by capillary forces. The rods are l=10 mm long and have diameter d=1.5 mm. They were placed on the water surface of a Petri dish with 10 cm diameter. Subfigures (**a**–**d**) correspond to times t=0 s (the initial distribution of rods on the water), t=30 s, t=90 s, and t=180 s, respectively.

The aggregation of objects floating on a liquid surface has been studied for a long time and generated a considerable literature [1,2,3,4,5]. The driving factors of aggregation are capillary forces that are attractive for objects of the same type of material (hydrophilic or hydrophobic). For symmetric disk-shaped objects, capillary forces are central and decay exponentially [6,7,8,9]. In the case of other shapes, the strongest capillary attraction is observed in directions leading to the largest decrease in the surface energy. For rods, this occurs when they are parallel, and the force is perpendicular to their orientation. Capillary forces are short-ranged, so if the object packing is low, then the time needed to form a stable aggregate is long. The final metastable structure of the aggregate is densely packed with dislocations characterized by very long relaxation time. Figure 1 shows an example of aggregation for 20 polylactic acid rods (10 mm long and 1.5 mm diameter) inside a Petri dish of 10 cm diameter. For the initial state of randomly distributed rods (cf. Figure 1a) the metastable structure composed of a few linked raft-like aggregates appeared after 3 min of medium evolution.

In this paper, we report aggregation in a medium of higher complexity in which, parallel to capillary attraction, there are Marangoni flows and repulsion between objects’ forces related to surface tension modification by the dissipated surface-active molecules [10,11,12,13]. We expect the medium with complex interactions between individuals should produce aggregates with less trivial metastable structures than those densely packed structures observed for pure capillary forces. The experimental data should help to discover the relationship between an object’s shape and its ability to aggregate. Since both the capillary forces and the release rates of surface-active molecules depend on the shape, we can expect that they play a crucial role in times of aggregate formation and times of relaxation of their structures.

A camphor piece placed on a water surface is one of the well-known examples of self-propelled objects [14,15,16]. The self-propulsion of camphor disks has been studied for more than a century [17,18]. Under typical laboratory conditions, a piece of camphor located on a water surface is an open system in which molecules released from the source form a surface layer and are finally dissipated into the air. The mechanical effects associated with the presence of surface-active molecules can be explained through the surface tension that depends on the local value of their surface concentration c(x,y), where (x,y) describes the location on the surface. In the case of camphor, the surface tension γ drops from γ(c=0)∼72 mN/m for pure water to $\gamma (c_{sat})∼40$ mN/m for water covered with a saturated camphor surface layer.

The functional dependence of the water’s surface tension on c(x,y) can be approximated by [19]

(1) γ(c(x,y))=γ(0)/(1+Γ·c(x,y))≅γ(0)(1−Γ·c(x,y)).

where Γ is a constant.

The assumption that the energy of the medium can be approximated as the surface energy of water leads to the explicit formula for the force F acting on an object with the geometrical location defined by the time-dependent characteristic function $\chi_{\Omega }\left(x,y,t\right)$:

(2) $$F\left(t\right)={\;\int \int \;}_{\Omega }\left(\nabla \gamma (r,t)\right)\chi_{\Omega }\left(r,t\right)dr$$

Similarly, the torque acting on an object, calculated with respect to the vertical axis located at $r_{c}$, can be expressed as

(3) $$N\left(t\right)={\;\int \int \;}_{\Omega }\left(r-r_{c}\right)\times \left(\nabla \gamma (r,t)\right)\chi_{\Omega }\left(r,t\right)dr$$

Here, the operator × denotes the vector product in two dimensions, that is, $a\times b=a_{x}b_{y}-a_{y}b_{x}$ for $a=(a_{x},a_{y})$ and $b=(b_{x},b_{y})$. The formulae given above are consistent with the peripheral integration of the surface forces that act in the normal direction at the object’s periphery [16,20].

Formulas (2) and (3) return F=N=0 when the surface tension is constant around the object. The broken symmetry [21] of c(x,y) is the necessary condition for self-propulsion. Symmetry breaking can be realized in two ways. Objects can be intentionally designed for asymmetric dissipation of surface-active molecules, as with camphor boats, with the source located at the stern [22]. The elliptic camphor particles move with a velocity oriented perpendicularly to the major axis [23]. It can be anticipated that shape and motion are correlated because the source shape defines the resistance to its motion on the surface and determines the local outflow of surface-active molecules. No wonder research on the relationship between the symmetry and the geometrical shape of a solid, self-propelled object and the character of its motion has stimulated abundant scientific activity [23,24,25,26,27,28,29]. For symmetrical objects, fluctuation-induced symmetry breaking can occur. Assume an object is a source of surface-active molecules. Areas characterized by a low surface concentration can appear as the result of fluctuations in concentration resulting from the stochastic character of dissipation or evaporation of surface-active molecules. If the object moves from its original position, then initially, it shifts to the area characterized by large gradients of the surface tension. This significantly increases the object’s speed. Once the object starts moving, its motion is maintained as long as the surface tension in front of it is higher than behind it. This is true for a low density of camphor- (or camphene-) propelled objects because under typical laboratory conditions, camphor molecules evaporate quickly; hence, the water’s surface tension recovers in a short time. As a result, self-propelled motion can be observed for a long time, exceeding 1 h, until all active molecules are dissipated. However, in the case of many sources, the concentration of surface-active molecules on the water surface can be high, gradients low, and speeds of objects small.

As long as the surface-active molecules are released, the repulsive forces and torques defined by Equations (2) and (3) are much stronger than the capillary attraction, especially at distances exceeding 1 cm. However, at very long times, exceeding a few hours, when surface-active molecules finally dissipate, capillary forces become dominant, and highly packed structures are formed. The evolution towards highly packed structures is clearly seen in experiments with droplets [30] and camphor gel disks [31].

For typical experiments on aggregation of self-propelled objects, we need a number of similar (preferably identical) objects with the required shape. However, making camphor pieces of the required shape is difficult, especially when many objects with the same shape are needed. A few techniques can be used to transform camphor grains into objects of defined shape. The most popular is pressing camphor granules together in a pill maker, but this technique mainly applies to small camphor disks [22]. An inert porous support material, like a paper membrane [32] or agar gel [33,34], can be impregnated with a camphor solution. Still, this technique is not ideal because it is hard to control the amount of surface-active agent, which may lead to its non-homogeneous distribution in the membrane. Moreover, the saturated objects contain smaller amounts of camphor than pills, limiting surface activity to minutes [27].

In this respect, the camphene–camphor–polypropylene plastic seems to be ideally suited for experiments on surface aggregation. This plastic was discovered during our research on novel materials for experiments on self-propelled motion on water surfaces. Under typical laboratory conditions, camphor is a solid formed in tiny granules. The camphene molecule is similar to camphor, and it exhibits a similar, albeit reduced, surface activity. At room temperature, camphene is waxy and very malleable. The melting temperature of camphene is ∼51 °C [35], much lower than that of camphor (∼175 °C [36]). We observed that at temperatures above 130 °C camphor easily dissolves in molten camphene in a 1:1 mass ratio. The camphene–camphor mixture transforms to a sticky wax after it is cooled down to room temperature [37]. It can be cut to the required shape. In the mixture, camphene plays the role of a solvent. The wax hardness increases with the mass fraction of camphor. At yet higher temperature, above 200 °C, polypropylene can be dissolved in the mixture of camphene and camphor. Thus, it appears as the next component of camphene–camphor wax [38]. Polypropylene forms an interesting microporous skeleton [39] and changes the mechanical properties from waxy to plastic. Moreover, it regulates the dispersion of surface-active molecules, prolonging the self-propelled motion of objects made of it for many hours. There are a few arguments for using the camphene–camphor–polypropylene plastic: First, we demonstrated that the motion of disks made of this material continues for 3 h without a significant decrease in speed (cf. Figure 15 in [38]). Next, the surface activity of objects made of camphene–camphor–polypropylene plastic does not significantly depend on the weight ratios of the compounds. Finally, the material can be easily processed and shaped on most clean substrates. Rods can be made by cutting long camphene–camphor–polypropylene filaments obtained by pressing hot plastic out from a syringe through a hole with the selected diameter, as described in [38]. The ability to generate rods encouraged us to use them as an example of aggregation of objects with high anisotropy.

**Figure 2:**
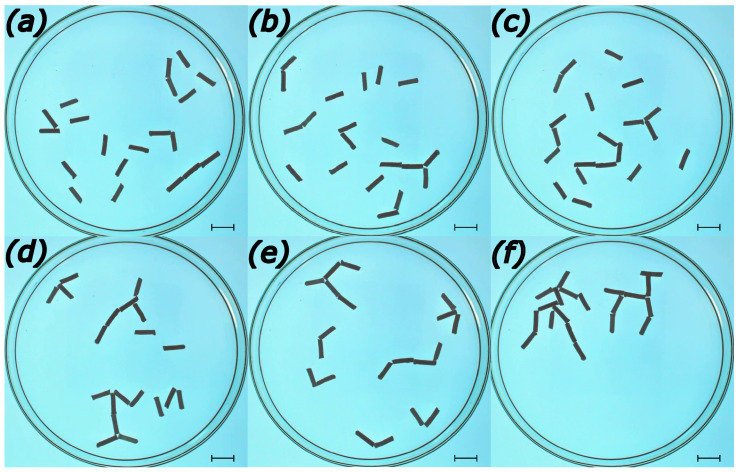
An example of aggregation in a system of 20 rods inside a 12 cm Petri dish. Each rod is l=10 mm long and has d=2 mm diameter. Subfigures show the positions of rods at different times: (**a**) shortly after rods were placed on the water surface (t=0); (**b**–**e**) correspond to times $t_{b}=6$ s, $t_{c}=26$ s, $t_{d}=73$ s, and $t_{e}=90$ s. The surprising cave-art style figure of a hunter chasing an animal (**f**), here shown for $t_{f}=25$ min, was generated by self-aggregated roots not by a human hand. The movie illustrating the most important fragments of the evolution is included in the Supplementary Information as 140-first-30s.mp4 (the first 30 s of time evolution after all rods are placed on the water surface), 140-second-1m30s.mp4 (evolution in the time interval [30 s, 2 min]), 140-start12m-end14-30.mp4 (evolution in the time interval [12 min, 14 min 30 s]). Scale bars are 10 mm.

**Figure 3:**
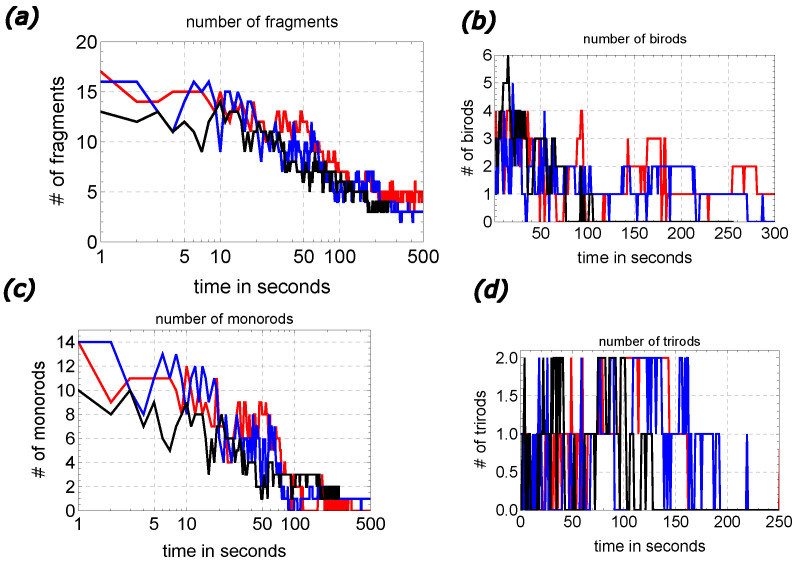
Qualitative analysis of the number of fragments as a function of time in systems of 20 self-propelled rods (l=10 mm, d=1.5 mm) aggregating inside 12 cm Petri dish: (**a**) the total number of fragments; (**b**) the number of birods; (**c**) the number of monorods; (**d**) the number of trirods. The results were obtained from the time evolution leading to metastable structures illustrated in Figure 4b,c,e (red, blue and black curves, respectively).

The initial location of the rods has a strong influence on their future evolution if repulsive interactions are absent and capillary attraction dominates (cf. Figure 1). Self-propelled rods are very active at the beginning of the experiment (cf. Figure 2a,b), when the surface concentration of active molecules is low and gradients high. Therefore, for a low surface density of rods, the regular initial structure is completely disturbed, and different distributions show similar time evolution (cf. Equations (12) and (13) and Figure 3). In our experiments, rods were intentionally distributed at random. In some, we located rods one by one on the surface. Such a method gives better control of the initial disk location, but it can be applied to a small number of rods only. The role of initial distribution increases with the surface density of rods because the mean free path of a rod becomes shorter, and the initial local configuration has a stronger influence on the evolution.

Let us also notice that a single symmetrical self-propelled object requires an induction time before it starts to move. During this time, fluctuations in the initial symmetrical distribution of surface-active molecules develop around the object and non-zero net force appears. Such an induction time is not observed in many-object systems because the random initial distribution of symmetrical objects can generate non-homogeneity required to initiate the motion. The time evolution of systems containing 20 rods that were 10 mm long and had a diameter of 1.5 mm on a water surface in a Petri dish with a diameter of 12 cm is shown in Figure 2. The rods are made of 10% polypropylene, 45% camphene, and 45% camphor. Movies showing the most interesting fragments of the rod aggregation are included in the Supplementary Information.

**Figure 4:**
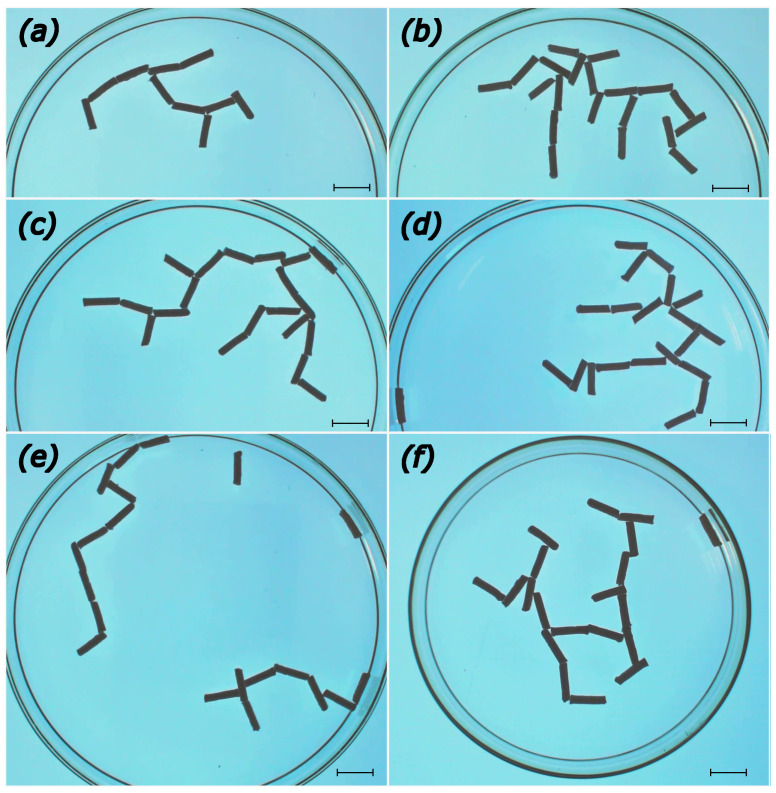
The metastable aggregates observed at the end of experiments. Subfigures (**a**–**c**,**e**) correspond to times $t_{a}=63$ min, $t_{b}=60$ min, $t_{c}=65$ min, and $t_{e}=56$ min. The structure shown in (**d**) ($t_{d}=12$ min) aggregated after dispersing an existing structure to single rods, i.e., 12 min after a concluded 60 min experiment. The structure in (**f**) ($t_{f}=16$ min was formed in a smaller Petri dish with the diameter d=10 cm. The freeze frames depicted were cropped in order to show the full extent of aggregates and all scale bars are 10 mm.

We can distinguish a few phases in the medium evolution. At the very early stages of evolution, the system is composed mainly of monorods and occasional birods. A single trirod seen in Figure 2a can be regarded as a rare occurrence. At the early stages of evolution (Figure 2a,b) the monorods combine to make birods joined at different angles at the rod ends. Next, the objects concatenate and form a few weakly joined rod aggregates (Figure 2c–e). However, due to there still being a high concentration of surface-active molecules these structures are not stable and can break into smaller fragments or combine into other shapes. At this stage, corresponding to t<100 s (Figure 2d,e), monorods disappear completely. The number of birods fluctuates with the decreasing maximum. The observed speed of fragments decreases due to saturation of the water surface with surface-active molecules and a larger mass of formed fragments. Unlike the aggregation resulting from capillary forces, we do not observe clusters in which rods are attached in parallel. As the result of repulsive interactions, the observed density of the aggregates is much lower than those in Figure 1. Moreover, end-to-end contacts between objects are dominant. At longer times, exceeding 10 min, we observed large structures that grouped almost all rods. The surprising cave-art style graffiti of a hunter chasing an animal [40], illustrated in Figure 2f for $t_{f}=25$ min, appeared as a self-made aggregate of rods 13 min after the rods were placed on the water surface. The reader may be surprised that it was generated without any human help; we believe that if such a structure was found in a cave excavation it would be attributed to human hands. The hunter chased the animal for over 20 min, rotating clockwise around the dish wall, and the geometry of both aggregates remained stable. The probability of the self-generation of such an aggregate seems very low, and we wonder if it would be possible to repeat the evolution even if the rods were precisely located at their initial positions. At the end of the experiment, both hunter and animal combined to make another metastable structure, shown in Figure 4b.

## 2. The Theoretical Background of Models Describing Aggregation of Self-Propelled Objects

This section briefly describes the mathematical modeling of aggregation. Many papers report complex behavior in systems of self-propelled objects [14,41,42], but still mathematical models for many-object evolution are mainly limited to structureless objects described by Vicsek-type [43] dynamics [44]. Here, we discuss what equations can be used to simulate the evolution observed in experiments. Our attention is focused on conveying why the computational complexity of the problem goes beyond our numerical facilities. Nevertheless, we think the readers are interested in how the model can be constructed and what approximations are necessary to make it computable.

For a complete description of the aggregate dynamics, we have to know positions R(t) and orientations O(t) of all objects as functions of time. The velocities V(t) and angular velocities ω(t) can be obtained as time derivatives of positions and orientations. However, the phase space of variables in which the complete description of the system can be formulated is much larger and includes the time-dependent surface concentration of active molecules on the whole surface c(x,y,t), and the position-dependent surface flows $W_{2}\left(x,y,t\right)$. As we describe below, all these quantities are strongly coupled.

The Newtonian equations that include capillary and Marangoni interactions and viscous drag can describe the time-dependent positions of solid (non-deformable) objects. Schematically, such equations can be written as

(4) $$\begin{array}{cc} m\frac{\partial^{2}R}{\partial t^{2}} & =F(R(t),O(t),c(x,y,t))+capillary\_forces(R(t),O(t),c(t))\\ \; & +viscous\_drag(R\left(t\right),O\left(t\right),V\left(t\right),W_{2}\left(x,y,t\right)) \end{array}$$

where *m* is the object mass. Here, the first term represents the driving force resulting from the non-homogeneous distribution of surface-active molecules, and it can be expressed by Equation (2) and the relationship (1). The capillary forces between solid objects on the surface of a liquid describe the change in energy as the result of the appearing intervening menisci [45]. They are short-ranged and decay exponentially, but their precise description is very complex and involves hard calculations [46]. In the case of two rods, the capillary forces depend not only on the distance but also on the relative orientation between the rods. Moreover, in the systems we consider, the surface tension is not constant but depends on the local concentration of surface-active molecules. As far as we know, the problem of capillary attraction of objects that dissipate surface-active molecules that can locally change the surface tension has not yet been considered in the literature. The last term is a viscous drag, describing the resistance felt by an object moving on a fluid surface due to the viscosity of the fluid. The drag depends on the size, shape, and speed of the object with respect to the fluid. Here, we anticipate that the main contribution to the drag term comes from the difference between $W_{2}\left(x,y,t\right)$ and V(t).

Similar equations with torques, instead of forces, can be used to calculate the changes in the orientations of the considered objects.

The surface concentration of active molecules c(x,y,t) can be calculated by considering the supply of active molecules from the sources, the transport on the surface, and evaporation combined with dissolution. The corresponding equation has the form

(5) $$\begin{array}{cc} \frac{\partial c}{\partial t} & =surface\_flow\left(c\left(x,y,t\right),W_{2}\left(x,y,t\right)\right)+molecule\_supply\left(R\left(t\right),O\left(t\right)\right)\left(x,y\right)\\ \; & -evaporation(R(t),O(t),c(x,y,t)) \end{array}$$

The surface flow term can be expressed as [47]

(6) $$surface\_flow\left(c\left(x,y,t\right),W_{2}\left(x,y,t\right)\right)=\nabla \cdot \left(c\left(x,y,t\right)\;\;W_{2}\left(x,y,t\right)\right)$$

Camphene is practically insoluble in water [35], whereas camphor’s solubility is small enough to neglect it if compared with evaporation [36]. The evaporation of surface-active molecules can be approximated by a single-molecule process. Thus, the rate of this process should be proportional to c(x,y,t), provided that a given region of space is not covered by the object. The arguments given above lead to

(7) $$evaporation\left(R\left(t\right),O\left(t\right),c\left(x,y,t\right)\right)=b\;\;c\left(x,y,t\right)\;\;\left(1-\sum_{i}\chi_{\Omega (R_{i}\left(t\right),O_{i}\left(t\right))}\left(x,y\right)\right)$$

where the summation is performed over all objects and $\chi_{\Omega (R_{i}\left(t\right),O_{i}\left(t\right))}$ denotes the characteristic function of object #i characterized by $R_{i}\left(t\right)$ and $O_{i}\left(t\right)$ and *b* is the rate constant.

We can describe the molecule_supply to the water surface assuming that its rate is equal for all points of the source that have contact with water:

(8) $$molecule\_supply\left(R\left(t\right),O\left(t\right)\right)\left(x,y\right)=a\;\;\sum_{i}\chi_{\Omega (R_{i}\left(t\right),O_{i}\left(t\right))}\left(x,y\right)$$

where *a* is the local supply rate.

The flow $W_{2}\left(x,y,t\right)$ is the surface (boundary) value of the 3-dimensional flow $W_{3}\left(x,y,z,t\right)$ induced in the water volume by the Marangoni forces [48] and the motion of hard objects on the water surface. The set of corresponding Navier–Stokes equations is the most complex numerical part of the model. Fortunately, a realistic approximation for the time evolution can be obtained if the real hydrodynamic flow in the transport equation for surface-active molecules is approximated by a reaction–diffusion equation [47]:

(9) $$\frac{\partial W_{2}}{\partial t}=flow\_relaxation\left(V\left(t\right),W_{2}\left(x,y,t\right)\right)-\eta_{2}\;\;W_{2}\left(x,y,t\right))+D_{\eta }\;\nabla^{2}\;W_{2}\left(x,y,t\right))$$

Within this approximation, the flow relaxation term is equal to

(10) $$\begin{array}{cc} flow\_relaxation(V\left(t\right),W_{2}\left(x,y,t\right)) & =\\ \eta_{1}\;\;\left(W_{2}\left(x,y,t\right)-velocity_{(R_{i}\left(t\right),O_{i}\left(t\right))}\left(x,y\right)\right)\; & \;\chi_{\Omega (R_{i}\left(t\right),O_{i}\left(t\right))}\left(x,y\right) \end{array}$$

The second term in Equation (9) takes into account the viscous effect in the vertical direction and the last term describes the relaxation of $W_{2}\left(x,y,t\right)$ on the surface.

The concept of an effective diffusion constant [49] can be also used to simplify Equation (5) to the form

(11) $$\begin{array}{c} \frac{\partial c}{\partial t}=\nabla \left(D\left(x,y\right)\nabla c\right)+molecule\_supply\left(R\left(t\right),O\left(t\right)\right)\left(x,y\right)-evaporation\left(R\left(t\right),O\left(t\right),c\left(x,y,t\right)\right) \end{array}$$

Here, D(x,y) is the effective diffusion coefficient of the surface-active molecules at the water surface adjusted to match experimental data [50]. In the case of two active molecules, i.e., camphor and camphene in the reported experiments, separate equations for each substance are needed. Moreover, the formula relating the surface tension with the surface concentrations (cf. Equation (1)) becomes a function of two variables. The set of Equations (4), (9), and (11) combined with the equation for orientation, makes the basis for an approximate numerical model that simulates hard-object aggregation. The calculation of capillary forces seems to be the most computationally demanding part of it. Therefore, even after the approximations are introduced, the numerical solution for many moving objects represents a hard numerical problem.

The evolution equations listed above show some analogy with quantum problems because the equation for object dynamics involves information about a spatiotemporal function that characterizes the medium. In the quantum case, it is the density of considered particles, for example, in the Gross–Pitaevskii equation [51,52]. Here, the influence of the medium is included in concentrations of surface-active molecules. It is also worth mentioning that numerous surface vortices can be observed during experiments with self-propelled objects of non-trivial shapes. The question of whether medium vorticity has any influence on aggregation analogous to what is reported in quantum systems remains open. The presented experimental medium seems suitable for future studies on this problem.

## 3. Experimental Aggregation of Self-Propelled Rods and Basic Properties of Their Interaction

In this section, we present selected experimental results that can help formulate a qualitative model of aggregation and the appearance of metastable structures.

In the Introduction, describing the evolution illustrated in Figure 2, we argued for different phases of aggregate formation. This information can be quantitatively analyzed by measuring the number of aggregates of different sizes. The results for aggregation of 20 rods (l=10 mm, d=1.5 mm) are shown in Figure 3. We compare the results for the evolution shown in Figure 2 (metastable structure in Figure 4b, red curve) with the evolution with metastable structures shown in Figure 4c (blue curve) and Figure 4e (black curve).

Surprisingly, aggregates starting from very different initial conditions share similar time evolution, provided the statistical sample is large enough. In both cases, the number of monorods ($n_{m}\left(t\right)$; cf. Figure 3c) remained stable at the level of 50% of all rods used for the first 10 s of the experiment. Next, the number of single rods decayed logarithmically in time:

(12) $$\begin{array}{c} n_{m}\left(t\right)≅n_{m}\left(t=5\;s\right)-\alpha_{m}\left(\log_{10}\left(t\left[s\right]\right)-1\right) \end{array}$$

with $\alpha_{m}∼9$ for t∈[10,100] s. Finally, in both experiments, all monorods disappeared after 200 s of medium evolution. A relationship similar to Equation (12),

(13) $$\begin{array}{c} n_{f}\left(t\right)≅n_{f}\left(t=5\;s\right)-\alpha_{f}\left(\log_{10}\left(t\left[s\right]\right)-1\right) \end{array}$$

with $\alpha_{f}∼8$ gives a good approximation of the total number of separate fragments $n_{f}$ for t∈[10,200] s. Of course, the parameters $\alpha_{m}$ and $\alpha_{f}$ are not universal and they depend on rod density and, for the small dishes, on their sizes.

The number of birods illustrated in Figure 3b shows a maximum in the time interval [20,70] s, and then gradually decreases in time, with occasional fluctuations of smaller and smaller amplitude. The time evolution of the trirod number (cf. Figure 3d) has a fluctuating character, but in both cases, such aggregates disappear after 250 s. This indicates that at this time, due to the dispersion of surface-active molecules, large aggregates are bonded strongly enough to remain stable.

The surprising coincidence between the time evolution seen in different experiments indicates the necessity of future studies on aggregates with a large number of fragments and similar packing fractions. The results should clarify a macroscopic model of the process and relationships between a number of fragments of different lengths. This could help to understand the most probable channels of large clusters separating to smaller fragments.

Figure 4 illustrates a number of metastable aggregates observed after circa 1 h of evolution in a system of rods. We can see that even when the times of aggregation were almost 2 orders of magnitude longer than those in Figure 1, the structures are still not densely packed. Almost all structures except Figure 4f self-aggregated in a Petri dish with a diameter of d=12 cm. In all experiments except case (a), we used 20 rods. They correspond to aggregation times of $t_{a}=63$ min, $t_{b}=60$ min, $t_{c}=65$ min, and $t_{e}=56$ min. In almost all cases, the rods formed a single aggregate. The structure shown in (d) ($t_{c}=12$ min) aggregated after dispersing an existing structure to single rods, i.e., 12 min after a concluded 60 min experiment. We expect in this case the saturation of water with camphor is much higher than at the initial stage of the other experiments and this significantly changes the character of the initial stage of evolution. The structure shown in (f) ($t_{e}=16$ min) was formed in a smaller Petri dish with a diameter d=10 cm. Therefore, as can be anticipated, the process of aggregation accelerated at a higher rod density.

But how do we describe the observed metastable aggregates? The obvious method is to list the coordinates of the ends for all rods. It seems that 2-digit accuracy for each coordinate should be sufficient for 1 mm precision.A rough estimation shows that nine characters are needed for a single rod (2∗(2+2) digits for coordinates and one character marking the end of the rod data). Thus, a 10-rod aggregate requires 90 characters to define it. This description is precise but does not seem practical. Metastable aggregates can rotate and vibrate; this description corresponds to a specific observation time.

However, we can reduce the complexity of the description. Let us consider a pair of joint rods. A possible simplified coding can be based on the assumption that the first rod is directed south *S*. The location of the second rod can be described by the position of its connection with the first one and the angle between the first and the second one. Next, the procedure continues with the second rod being treated as the new first one. Combined with information about the chain branching, such information allows for a complete description of the aggregate. In practice, we do not need a high precision in angle definition because the aggregates are flexible and vibrate, so the angles can fluctuate within a certain range. A possible simplified angle coding can be achieved with a relative bearing between rods using the compass notation. The branching of the aggregate can be indicated by brackets. in addition to direction symbols, we need a symbol to indicate T-shaped connections, with bracket structures {{s1},{s2},{s3}}, where s1, s2, and s3 denote strings starting from the left, central, and right sites of the upper bar. For example, the aggregate shown in Figure 4b can be coded as S_E_N_{{N_N},{E_N_{N_E,E}}}. On the other hand, the left chain in Figure 4f is represented by the string S_W_N_N_E_N_W_T{{},{},{N_N}}.

As seen, this method allows for coding of an aggregate with fewer symbols than the orthodox one.

**Figure 5:**
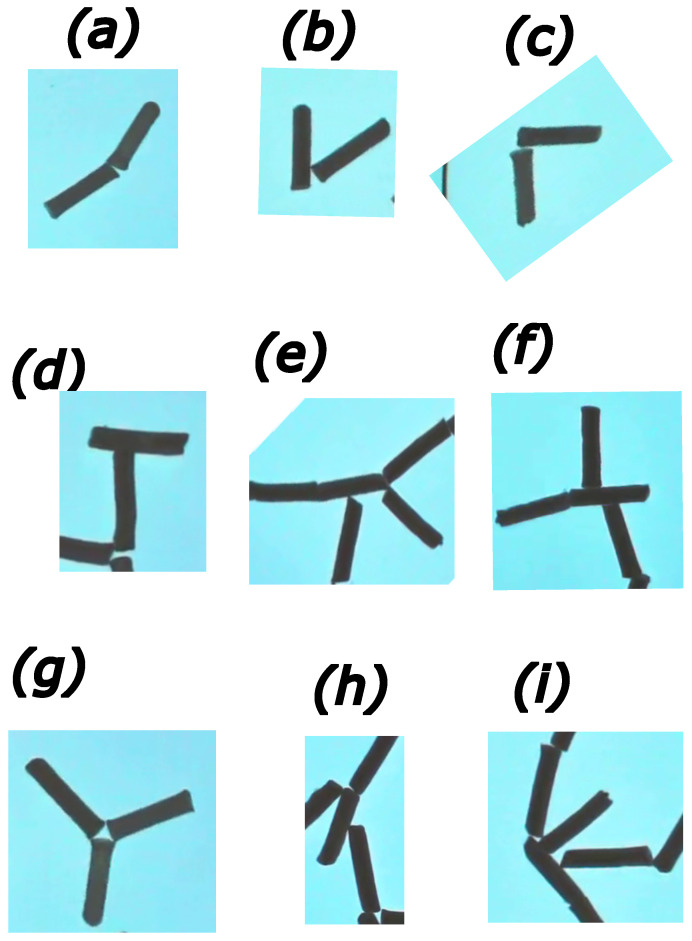
Simple fragments of aggregate that can be used as an alphabet to code a large structure of rods. End-to-end connections between two rods: (**a**)—*I* junction, (**b**)—*V* junction, and (**c**)—Γ junction (there is also a rotated version ℸ). End-to-center connections between two rods: (**d**) *T* junction; (**e**,**f**) complex branching following the *T* junction corresponding to T{{1},{},{V}} and T{{1},{1},{}}; (**h**) λ–connection. (**g**) End-to-end connections between three rods–the *Y* junction. (**i**) A complex connection of rods needing a separate symbol.

An alternative method of aggregate description is based on a list of frequently observed connections between rods. This technique uses the idea of information coding, relating the most common strings with simple symbols [53]. Here, we can apply the idea to classify connections between rods. We observed that the rods can form end-to-end and end-to-center junctions. The parallel connection so common for aggregation with capillary forces (cf. Figure 1) does not appear. Most of the observed connections are between two rods. Connections involving three rods are less frequent. We also observed occasional connections between four rods; the number of them is small, still special symbols should be introduced to code them. The examples of symbol coding are shown in Figure 5. The most common element of aggregates is an end-to-end junction of two rods at an angle close to straight (*I* junction). Another common junction of two rods is a junction at a right angle (Γ junction or its rotated version ℸ). Yet another junction seen in metastable aggregates is the joining of two rods at an acute angle (*V* junction). The junctions between two rods where one rod joins the center of the other are less frequent. If rods join at a right angle, we call it a *T* junction, and when the angle is acute, we name it λ. The end-to-end connections can also occur between three rods, giving a *Y* junction. Junctions *T*, *V*, *Y*, or λ can lead to aggregate branching. We can use the previously described idea of bracketing the parts. The symbol T{{s1},{s2},{s3}} denotes strings originating from the left, central, and right positions on the upper bar. A single rod attached should be coded with separate symbols, for example, 1. Similarly we can use V{{s1},{s3}} and Y{{s1},{s3}} to code branches aggregating from left and right arms, respectively.

The analysis of metstable aggregates observed in our experiments gives the following probabilities of junctions: $P_{I}∼0.35$, $P_{\Gamma }∼0.32$, $P_{T}∼0.16$, $P_{V}∼0.08$, and $P_{Y}∼P_{\lambda }∼0.03$. Knowledge of these probabilities allows us reverse the problem. We can simulate aggregates by generating random strings of symbols with the probabilities given above and decoding the corresponding geometrical structures of the rods. Would this represent a real aggregate? Keeping in mind the variety of structures we generated, we could be lucky and find a matching one, but the convincing answer to this question can be given after collecting more experimental data. For example, we still do not know if there are any symbol correlations in strings representing real aggregates.

## 4. Conclusions and Perspectives

**Figure 6:**
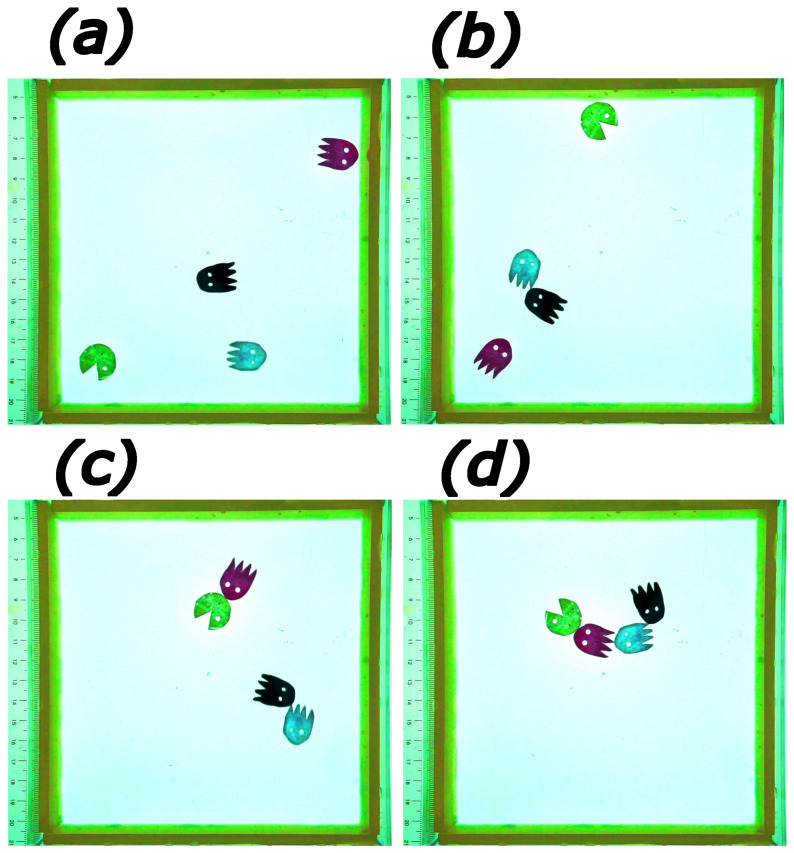
Aggregation of PacMan [54] characters made of camphene–camphor–polypropylene plastic floating on water surface inside square-shaped 15×15 cm area. Subfigures (**a**–**d**) correspond to the times 8, 17, 23, and 26 s. The movie illustrating the time evolution can be watched on YouTube ([56]).

In the paper, we highlighted a recently discovered camphene–camphor–polypropylene plastic as a material useful for studies of aggregation of self-propelled objects on a water surface. The material is perfectly suited for such experiments because it can be easily formed into the required, complex shapes. The ability to produce many objects with standard shapes is important to study the effect of object density on aggregation. In our experiments, we used it to investigate the interesting aggregation of self-propelled rods. We plan to extend our research by including the influence of the length/diameter ratio on aggregation. Moreover, we plan to introduce an additional complexity to the problem by considering a mixture of rods of different sizes. Of course the material can be cut to represent objects of more complex geometry. Figure 6 illustrates the evolution of PacMan [54] characters made of this plastic. The characters are circa 15 mm in size and move on a square water area of 15×15 cm. Objects characterized by more specific shapes can be used to visualize key-and-lock matching between molecules and study its role in the formation of large complexes [55].

The properties of the camphene–camphor–polypropylene material are robust with respect to its components, and both plasticity and self-propelled motion are observed within a large range of weight fractions. By changing the composition we are able to modify the release rates of surface-active molecules and control the ratio between capillary and Marangoni interactions. This can slow down or speed up times of transition towards a densely packed aggregate. Also, dyes are expected to moderate the release of surface-active molecules [30,57,58]. Finally, the object mass can modify the strength of capillary attraction, and this parameter can be regulated by the addition of metal particles to the plastic when it is prepared.

In our experimental studies, we were concerned with rods floating on a water surface inside a Petri dish. However, the geometry of the water area where the objects self-assemble can also influence the process of aggregation. Such effects are anticipated in biological cells characterized by a high density of different organs. In experiments on aggregation with self-propelled plastics, geometrical constraints can be introduced using a thick silicon foil that does not affect self-propulsion by camphor or camphene molecules.

Finally, we foresee potential applications of aggregation with self-propelled objects for unconventional computation. Let us assume that fragments of the aggregate can be mapped into symbols. The aggregate can be regarded as a string of them, as we described in the previous section. An aggregating system can be regarded as a solver of an optimization problem where energy minimization is the task function. If so, aggregation with self-propelled objects can be seen as an annealing optimization in which the decreasing concentration of dissipated surface-active molecules plays a similar role to decreasing temperature [59]. Delving further, the considered medium could reveal closer analogies with quantum vortices [51,52].

## 5. Materials and Methods

The detailed procedure for making camphene–camphor–polypropylene plastics was described in [38]. Here, we just give a short description. Samples at different weight ratios of compounds were prepared by weighing of commercially available (1R)-(+)-Camphor (98% purity, CAS: 464-49-3, Sigma-Aldrich, Darmstadt, Germany), camphene (95% purity, CAS: 79-92-5, Sigma-Aldrich, Darmstadt, Germany), and polypropylene in the form of pellets (CAS:9003-07-0, Sigma-Aldrich, Darmstadt, Germany, product number 427861) in a 50 mL beaker containing a magnetic stirrer, and covering it in order to prevent excessive evaporation. The mixture was placed on a hot plate set to 250 °C while stirring until all the polypropylene was dissolved in the liquid camphene–camphor mixture. The dissolution took between 20 and 40 min, depending on the ratio of components. The liquid could then be poured into a vessel lined with nitrile, latex, or silicone sheets, left to solidify, and finally cut to the required shapes. The rods used in the reported experiments were made of plastic composed of 10% polypropylene, 45% camphene, and 45% camphor as the weight ratio. While hot and soft at ca. 60 °C, this material was pressed out from a syringe through a hole with the selected diameter. The obtained long camphene–camphor–polypropylene filaments were cut into shorter rods of the lengths required for the experiments.

## Data Availability

The data presented in this study are available on request from the corresponding author.

## Supplementary Materials

The following supporting information can be downloaded at: https://www.mdpi.com/article/10.3390/e26110980/s1, 140-first-30s.mp4 (the first 30 s of time evolution after all rods are placed on the water surface), 140-second-1m30s.mp4 (evolution in the time interval [30 s, 2 min]), 140-start12m-end14-30.mp4 (evolution in the time interval [12 min, 14 min 30 s]) cf. Figure 2.
